# Antibacterial activity and phytochemical components of leaf extract of *Calpurnia aurea*

**DOI:** 10.1038/s41598-023-36837-3

**Published:** 2023-06-16

**Authors:** Yared Wasihun, Habtemariam Alekaw Habteweld, Kassahun Dires Ayenew

**Affiliations:** 1 Department of Internal Medicine, Ras Desta Damtew Memorial Hospital, Addis Ababa, Ethiopia; 2 Department of Pharmacy, Asrat Woldeyes Health Science Campus, Debre Berhan University, Debre Berhan, Ethiopia

**Keywords:** Biological techniques, Drug discovery, Microbiology

## Abstract

Local Ethiopians use *Calpurnia aurea* to treat skin infections. However, there is no adequate scientific confirmation. The aim of this study was to evaluate the antibacterial activities of the crude and the fractionated extracts of *C. aurea* leaves against different bacterial strains. The crude extract was made by maceration. The Soxhlet extraction method was used to obtain fractional extracts. The antibacterial activity against gram positive and gram negative American Type Culture Collection (ATCC) strains was performed using the agar diffusion technique. The minimum inhibitory concentration was determined through the microtiter broth dilution method. Preliminary phytochemical screening was done using standard techniques. The largest yield was obtained from ethanol fractional extract. Except for chloroform, which provided a relatively low yield when compared to petroleum ether, increasing the polarity of the extracting solvent improved the yield. The crude extract, solvent fractions, and the positive control showed inhibitory zone diameter, while the negative control did not. When used at a concentration of 75 mg/ml, the crude extract had similar antibacterial effects as gentamicin (0.1 mg/ml) and the ethanol fraction. The 2.5 mg/ml crude ethanol extract of *C. aurea* suppressed the growth of *Pseudomonas aeruginosa*, *Streptococcus pneumoniae*, and *Staphylococcus aureus*, according to the MIC values. The extract of *C. aurea* was more effective in inhibiting *P. aeruginosa* than the other gram-negative bacteria*. Fractionation enhanced the antibacterial effect of the extract.* All fractionated extracts showed the highest inhibition zone diameter against *S. aureus*. Petroleum ether extract had the greatest inhibition zone diameter against all bacterial strains. The non-polar components were more active compared to the more polar fractions. The phytochemical components discovered in the leaves of *C. aurea* included alkaloids, flavonoids, saponins, and tannins. Among these, the tannin content was remarkably high. The current results could provide a rational support for the traditional use of *C. aurea* to treat skin infections.

## Introduction

Medicinal plants serve as a complement or substitute for modern medical treatments^1,2^. Plants exhibit a wide range of structural and biological diversity. They contain phytochemicals^3,4^. These phytochemicals have a significant inhibitory effect on the growth of pathogens^5^.

A major challenge in global healthcare is the need for novel, effective, and affordable drugs to treat microbial infections, particularly in the world's developing countries. Some *Staphylococcus* and *Streptococcus* spp. are involved in the pathogenesis of respiratory and skin infections, together with *Pseudomonads* and representatives of *Enterobacteriaceae*, causing gastrointestinal, urogenital diseases and wound contamination. These microorganisms are practically resistant to older antibiotics^6^. To promote herbal medicine use, there is an urgent need to evaluate the therapeutic potential of medicines. Little work has been done to promote traditional medicine^7,8^.

*Calpurnia aurea* is a flowering plant in the Fabaceae family. The genus includes shrubs or small trees in or along the edges of forests in many parts of Ethiopia. It is widespread in Africa from the Cape Province to Eritrea. The southern part of India is also an additional habitat for the plant^9^.

A literature review reveals that all parts of *C. aurea* have been used for various human and animal diseases^4^. The plant is used treat different types of diseases such as amoebic dysentery, diarrhea, syphilis, tapeworm, leishmaniasis, trachoma, wound, tinea capitis, scabies, elephantiasis and various swellings in humans^10–13^. It has been found to have antibacterial^14–17^, antihyperlipidemic and antidiabetic^18,19^, antioxidant^20–23^, antihypertensive^24^, antiulcer^25^, antimalarial^26^ and anticancer activities^27^.

*C. aurea* has a significant amount of flavonoids, which have been shown to have potent antibacterial and antioxidant effects^21^. The aim of this study was to evaluate the antibacterial activity of crude and fractionated extracts of *C. aurea* leaves against different bacterial strains.

## Materials and methods

### Plant material collection and identification

*C. aurea* fresh leaves were collected from a wild source in Addis Ababa's Yeka sub-city after receiving permission from Ethiopian Biodiversity Conservation Institute, Addis Ababa, Ethiopia. Collection of the plant materials complies with relevant national and international guidelines and legislations. Plant identification and authentication was performed by Professor Ensermu Kelbessa from Addis Ababa University in Ethiopia. Voucher ID CA. 2013/01 was preserved for the voucher specimen in the herbarium of Addis Ababa University, Ethiopia. *C. aurea* fresh leaves were dried in a shed and ground with an electric grinder. After being sieved using sieve No. 45, the powder was kept in an airtight container until extraction.

### Preparation of the crude and fractional extracts

The extraction procedure was carried out using a previous method^28^. The 100 g of the powdered leaves were weighed on an analytical scale before being macerated with 70% ethanol in an Erlenmeyer flask. The powder was completely macerated for 9 days while being sometimes shaken on a rotary shaker (VWR DS-500; The Lab World Group, Boston, MA, USA). The extracted material was filtered through sieve mesh and Whatman no. 1. An extract filtrate was concentrated in a rotary evaporator (Buchii model R-200, Switzerland) at a temperature of 40 °C and 40 revolutions per minute (rpm).

Petroleum ether, chloroform, acetone, and ethanol were used to further fractionate the ethanol extract. For solvent fractionation, a scientific method was employed^29^. The 50 g of ethanol crude extract was weighed on an analytical balance and thoroughly dissolved in the beaker's 100 ml of 70% ethanol. For solvent partitioning, 100 ml of petroleum ether and 100 ml of dissolved ethanol crude extract were combined in a 250 ml separatory funnel. A clear and distinct layer between the two solvents appeared when the mixture in the separatory funnel was fixed to the stage pole. Once a transparent layer had developed, the petroleum ether portion was separated, and the ethanol portion was carefully transferred to a beaker. The petroleum ether partition was collected together for future concentration. While the remaining crude ethanol extract solution was subjected to evaporation in a rotary evaporator 40 °C and 40 rpm. Then, 80 ml of water was added to the concentrated crude ethanol extract to form an aqueous solution.

Similarly, 100 ml of the crude ethanol extract's aqueous solution was mixed with 100 ml of chloroform. A clear layer had to form between the aqueous solutions of crude ethanol extract and chloroform before the separatory funnel could be fastened to the stage pole. The portion of chloroform was kept at the bottom and collected first in the container, the portion of water was collected in another container. The chloroform part was separated for later concentration. The residual aqueous part of the crude ethanol extract was concentrated on a rotary evaporator.

In a separatory funnel, 100 ml of the concentrated aqueous component of the crude ethanol extract was mixed with 100 ml of acetone. A distinct layer between the aqueous fraction and the acetone fraction was formed. Aqueous and acetone fractions were collected in separate containers. The acetone fraction was concentrated in a rotary evaporator while the aqueous fraction was lyophilized by a lyophilizer (Operon, Korea vacuum limited, Korea).

### Standard drugs

As a positive control, gentamicin 10 μg disc was brought in from the Ethiopian Veterinary Drug and Animal Feed Administration and Control Authority, Veterinary Drug, and Animal Feed Quality Assessment Centre, Ethiopia. The substance ethanol was used as a negative control.

### Data analysis

The data were entered into an excel spreadsheet using the Statistical Package for Social Science (SPSS) version 23.

### Antibacterial activity test of the crude and fractional extracts

The agar diffusion technique was used to test the antibacterial activity against gram positive and gram negative ATCC microorganisms. Two gram positive bacterial strains (*S. aureus* and *S. pneumoniae*, ATCC55115 and ATCC53819) and two gram negative strains (*E. coli* and *P. aeruginosa*, ATCC56521, and ATCC57853, respectively) were employed. The complete medium of the offended microorganisms was grown on five percent red sheep blood agar plates. Growing bacterial suspension's turbidity was adjusted to 0.5 McFarland units by combining 0.5 ml of 1.75% (w/v) barium chloride dehydrate with 99.5 ml of 1% (v/v) sulphuric acid^28^.The inoculums of the various bacteria were streaked over the Mueller Hinton agar plates to completely cover the plates and become uniform after incubation. Using a sterile cork borer, 10 mm diameter wells were made on Mueller–Hinton plates. Each well was filled with 100 µl of the test substances and the plates were left at room temperature for two hours. The plates were then incubated for 24 h at 37 °C. The tube diffusion method was used to determine the minimum inhibitory concentration (MIC) of the crude and fractional extracts. The crude and fractional extracts were prepared at concentrations of 25, 50, 75, and 100 mg/ml by dissolving the dried extracts in chloroform (for chloroform and petroleum ether fractions) and ethanol (for acetone and ethanol fractions). Gentamycin was used as a positive control at a concentration of 0.1 mg/ml. 70% ethanol served as a negative control. The diameter of the zones of inhibition was used to calculate the antibacterial activity of all samples^29,30^.

### Preliminary phytochemical screening

*C. aurea* extracts were put through a preliminary phytochemical screening using the screening techniques previously described^1,14^. On a steam bath, 0.5 g of crude extract was mixed with 5 ml of 1% HCl to check for the presence of alkaloids. A few drops of Mayer's reagent were added to 1 ml of the filtrate, and a comparable amount of dragendorff's reagent was added to another. Preliminary evidence for the presence of alkaloids was taken into account when there was turbidity or precipitation with both reagents^31,32^. 0.5 g of crude plant extract was mixed with water in a test tube by employing the mobile phase of chloroform, glacial acetic acid, methanol, and water and the spraying reagent of vanillin-sulphuric acid for detection. A zone that is blue, blue violet, red, or yellow brown was regarded as a positive indicator of saponins^32,33^. For tannins,10 ml of distilled water and 0.5 g of crude extract were mixed together before filtering. The presence of blue, blue-black, green, or blue-green coloring or precipitation after adding FeCl3 reagent to the filtrate was interpreted as a sign for presence^32,33^. 0.5 g of *C. aurea* extract was mixed with 10 ml of benzene and filtered to check for anthraquinones. The filtrate was combined with a 10% ammonium hydroxide solution (5 ml), and the mixture was agitated. The presence of a pink, red, or violet color in the ammoniacal phase was a sign for anthraquinones. For the polyphenols, 3 drops of a solution containing 1 ml of 1% FeCl3 and 1 ml of 1% C_6_N_6_FeK_3_ were added to 2 ml of the crude extract. Green–blue color formation was interpreted as a sign of presence^32–34^. 2 ml of the crude extract's alcoholic solution and 4 drops of a 2% lead acetate solution were added to make a flavonoid solution. The appearance of a yellow or orange tint revealed the presence of flavonoids^31–33^.

### Ethical approval and consent to participate

Before the study began, the Institutional Review Board of Addis Ababa University in Ethiopia gave its approval. A letter of clearance was granted. Collection of the plant materials complies with relevant national and international guidelines and legislations.

## Results

The current investigation demonstrated the antibacterial activity of the leaf extract of *C. aurea*. The yields were sufficient; the largest yield was obtained from ethanol fractional extract. The lowest yield was obtained from chloroform fractional extract. Except for chloroform, which provided a relatively low yield when compared to petroleum ether, increasing the polarity of the extracting solvent improved the yield (Table 1).

**Table 1:** Percentage yields of crude and fractions of *C. aurea* leaf extract.

S.N.	Type of extract	Wt in gram	% yield w/w
1	Ethanol fractional extract	0.156	15.6
2	Crude extract	5.02	10.02
3	Petroleum ether fractional extract	0.0345	3.45
4	Chloroform fractional extract	0.0230	2.30
5	Acetone fractional extract	0.0750	7.50

The diameter of the inhibitory zone for crude and fractional extracts was measured by the agar well diffusion assay. The crude extract, solvent fractions, and the positive control showed the inhibitory zone diameter, while the negative control did not. All fractionated extracts showed the highest inhibition zone diameter against *S. aureus*. Petroleum ether extract had the greatest inhibition zone diameter against all bacterial strains. When used at a concentration of 75 mg/ml, the crude extract had similar antibacterial effects as gentamicin (0.1 mg/ml) and the ethanol fraction. Non-polar fractions showed more antibacterial activity than the polar fractions (Table 2).

**Table 2:** Antibacterial activities of different fractions of *C. aurea* against selected strains of bacteria.

Extract	Concentration mg/ml	Bacterial strain zone of inhibition (mm)			
		SA (ATCC55115)	EC (ATCC56521)	PA (ATCC57853)	Str (ATCC53819)
Petroleum ether	75	35	22	17	19
	50	31	21	17	17
	25	27	17	15	14
Chloroform	75	31	18	20	22
	50	25	17	18	21
	25	33	14	16	19
Acetone	75	31	18	20	20
	50	28	15	18	17
	25	22	12	15	14
Ethanol	75	29	19	20	19
	50	25	18	16	18
	25	21	15	14	16
Crude	75	30	18	22	22
	50	23	17	19	19
	25	19	15	17	15
Gentamycin	0.1	28	21	20	19

*SA* *S. aureus*, *EC* *E. coli*, *PA* *P. aeruginosa*, *Str* *S. pneumonia*.

The MIC of the crude extract against *P. aeruginosa*, *S. pneumoniae*, and *S. aureus* was 2.5 mg/ml (Table 3). The MIC for *E. coli* was higher than 2.5 mg/ml. Alkaloids, flavonoids, saponins, and tannins were among the phytochemical elements found in the leaves of *C. aurea*. Among these, the tannin content was remarkably high (Table 4).

**Table 3:** Minimum inhibitory concentration values of the crude (70% ethanol) extracts of *C. aurea* on the tested strains.

Plant species	Concentration mg/ml	Bacterial strain			
		Sa (ATCC6592)	Ec (ATCC57932)	Pa (ATCC47863)	Str (ATCC8938)
*Calpurnia aurea*	20	−	−	−	−
	10	−	−	−	−
	5	−	+	−	−
	2.5	−	+	−	−
	1.25	+	+	+	+
	0.625	+	+	+	+

*(+)* presence of growth, *(−)* absence of growth, *Sa* *Staphylococcus aureus*, *Ec* *Escherichia coli*, *Pa* *Pseudomonas aeruginosa*, *Str* *Streptococcus pneumoniae*.

**Table 4:** Phytochemical screening of crude (70% ethanol) extract of the leaves of *C. aurea* using chemical test methods.

Phytochemicals	Crude (70% ethanol) extract of *C. aurea*
Alkaloids	+
Saponins	+
Tannins	++
Anthraquinones	+
Polyphenols	−
Flavonoids	+

*−* negative, *++* strongly positive, *+* positive.

## Discussion

This study showed that the phytochemicals of leaf extract of *C. aurea* have antibacterial properties. Except for chloroform, which produced a poor yield when compared to petroleum ether, raising the polarity increased the yield. Ethanol produced the highest yield. This finding is consistent with Zelalem et al.^23^ and Mulatu^8^. However, a previous study noted an increase in yield when polarity declined^21^. 20–95% of the ethanol–aqueous mixture is frequently used in the herbal medicine industry to prepare extracts. Most known plant-based antibacterial compounds are soluble in organic solvents like ethanol^35^.

The existence of phytochemicals with therapeutic potential was checked in the leaves of *C. aurea*. Alkaloids, saponins, flavonoids, tannins, and but not polyphenols were found during testing. Among these, the content of tannins was remarkably high. Mulatu Getachew also noted the presence of alkaloids, tannins, flavonoids, and saponins^8^. A previous preliminary phytochemical analysis of the 70% ethanolic extracts of *C. aurea* seeds revealed the presence of substances such tannins, flavonoids, terpenoids, saponins, steroids, glycosides, and alkaloids^14^. According to phytochemical analysis and qualitative evaluation of the seeds and leaves of *C. aurea*, the seeds are richer in alkaloids and tannins than the leaves, but the leaves are richer in flavones and polyphenols^36^. The earlier phytochemical screening revealed that phenols and polyphenols were strongly present in the *C. aurea* root extract in both the ethanol and methanol extracts^37^. In contrast to alkaloids and anthraquinone, saponin, tannin, flavonoid, steroid, and terpenoid were detected in the qualitative phytochemical analysis of *Jatropha tanjorensis* ethanol, methanol, and aqueous leaf extracts^38^. Another qualitative investigation found that *Vernonia amygdalina* leaf extracts in both aqueous and ethanol included tannins, saponins, alkaloids, oxalate, phylate, flavonoids, steroids, and phenols. *Lawsonia inermis's* ethanol and aqueous leaf extracts' phytochemical analyses showed that the ethanol leaf extract contained glucose, saponins, sterols, and tannins, whilst the aqueous leaf extract solely contained flavonoids^39^.

As a result of plant genotypes, developmental phases, biotic variables (natural enemies, competitors, or mutualists), soil types and components, the time of year of collection, and geographic locations, secondary compound variation may also exist within a species breed^40,41^. The use of different extraction methods and solvents, as well as experimental error, may also contribute to this disparity.

Organisms utilized in this antibacterial activity test are frequently associated with both primary and secondary infectious skin problems^6^.

The current findings showed that *C. aurea* has antibacterial activity against all strains that was comparable to gentamycin at a concentration of 75 mg/ml. According to Tadeg et al., *C. aurea* has the same antibacterial effects as gentamycin at a concentration of 100 mg/ml^16^. When *C. aurea* was used against *S. aureus* compared to the positive control, antibacterial activities were greater, which is consistent with the findings of Adedapo et al.^22^. The antibacterial activity of the plant under test showed the positive correlation between concentration and the zone of inhibitions. This outcome is in line with earlier researches^8–11^.

In the current study, *C. aurea* exhibited stronger antibacterial action against gram positive than gram-negative bacteria. *S. aureus* was the most sensitive bacterial test strain. The least susceptible, however, was *E. coli*. This contradicts the argument presented by Umer et al., in^13^. According to Umer et al., the extract of *C. aurea* had the highest antibacterial activity against *S. typhi* and *E. coli* among the investigated bacteria.

According to reports, *E. coli* become resistant to many antibiotics. The sensitivity difference between gram-positive and gram-negative bacteria may be attributed to their differing morphological make-up. Compared to gram-positive bacteria, gram-negative bacteria frequently exhibit higher levels of resistance^42,43^.

The non-polar components were more active in our investigation compared to the more polar fractions. The antibacterial effects were seen to decrease as the polarity increased which indicating that the extract's active ingredients are concentrated more in the non-polar parts.

The 2.5 mg/ml crude ethanol extract of *C. aurea* suppressed the growth of *P. aeruginosa*, *S. pneumoniae*, and *S. aureus*, according to the MIC values. The extract of *C. aurea* was effective in inhibiting *P. aeruginosa* than the other gram-negative bacteria at 2.5 mg/ml. The extract had MIC value of greater than 2.5 mg/ml against *E. coli*. Meles et al.^5^, Elisha et al.^6^, Mulatu^8^, Bogale^9^ and others have reported the antibacterial activities of *C. aurea.*

The present findings are suggestive of the potential use of *C. aurea* for the treatment of skin and systemic infections.

## Conclusions

*Calpurnia aurea* showed different antibacterial activity profiles against the tested strains. *S. aureus* was the most vulnerable. *E. coli* being the least sensitive. The plant was tested positive for alkaloids, saponins, flavonoids, and tannins in a preliminary phytochemical screening analysis. The outcome of this study has demonstrated the potential of the plant for treating skin and systemic infections. More research on plants may be done regarding quantitative phytochemical analysis of the most antibacterial fractional extract for tannins, alkaloids, flavonoids, saponins, etc., for a comprehensive comparative study; or chemical characterization of the most active fractional extract by LC–MS/MS analysis, and/or activity-guided chromatographic isolation of compounds from the most active fractional extract, followed by NMR and MS characterization to identify the antibacterial compound(s). Additionally, research on the safety of plant extracts in both animals and human is necessary.

## Data Availability

All the data are contained in this paper. Kassahun Dires Ayenew, the corresponding author of this manuscript, will supply more information upon reasonable request. Email: kassh2009@gmail.com.

## Abbreviations

ATCC: American type culture collection
*E. coli*: *Escherichia coli*
*P. aeruginosa*: *Pseudomonas aeruginosa*
*S. aureus*: *Staphylococcus aureus*
WHO: World Health Organization
